# CCNE1 expression in high grade serous carcinoma does not correlate with chemoresistance

**DOI:** 10.18632/oncotarget.19272

**Published:** 2017-07-15

**Authors:** Stav Sapoznik, Sarit Aviel-Ronen, Keren Bahar-Shany, Oranit Zadok, Keren Levanon

**Affiliations:** ^1^ Sheba Cancer Research Center, Chaim Sheba Medical Center, Ramat Gan 52621, Israel; ^2^ Department of Pathology, Chaim Sheba Medical Center, Ramat-Gan 52621, Israel; ^3^ The Talpiot Medical Leadership Program, Chaim Sheba Medical Center, Ramat Gan 52621, Israel; ^4^ Sackler Faculty of Medicine, Tel-Aviv University, Ramat Aviv 69978, Israel

**Keywords:** CCNE1, ovarian cancer, predictive biomarker, chemoresistance, neoadjuvant chemotherapy

## Abstract

Delayed diagnosis of ovarian cancer, as well as high recurrence rates and lack of personalized therapy options, are among the causes for poor survival figures. Much effort is made towards developing new therapeutic possibilities, however predictive biomarkers are still unavailable. CCNE1 amplification, occurring in ∼20% of the high grade serous ovarian tumors, was previously proposed as a marker for platinum resistance and poor prognosis as well as for CDK2 inhibition. The current study aimed to examine the role of CCNE1 positive-immunostain as a predictor of first-line taxane-platinum chemoresistance. We evaluated matched pre- vs. post-neoadjuvant chemotherapy tumor samples and correlated the degree of pathological response to treatment with CCNE1 expression levels. Our results indicate that CCNE1 immunohistochemistry does not predict taxane-platinum chemoresistance in ovarian cancer patients. Further research is required in order to enable personalized adjuvant treatment, in cases where poor pathological response is achieved after the neoadjuvant phase.

## INTRODUCTION

Cyclin E1 (CCNE1), together with its catalytic subunit CDK2, has a central role in regulating cell cycle processes, assuring timely control of DNA replication and chromosome segregation. The complex induces S-phase entry as it activates S-phase specific genes and facilitates the formation of DNA replication complexes [1–3].

Overexpression of CCNE1 results in deregulation of the G1-S checkpoint and might predispose for the development of malignancy. Indeed, genomic amplification of the CCNE1-encoding 19q12-q13 locus was detected in a variety of human cancers including esophageal, gastric, lung, endometrial and ovarian [4, 5]. *In vitro* studies have shown that CCNE1 overexpression leads to reduced number of competent pre-replication complexes, which results in incomplete DNA replication followed by chromatid nondisjunction events and aneuploidy [6]. Such increased genomic instability might synergize with mutations of tumor suppressor genes and further fuel tumorigenesis.

*CCNE1* amplification occurs in ∼20% of high grade serous ovarian cancer (HGSOC) [7, 8]. It is one of the most frequent genetic alternations characterizing the disease, and is considered an early ‘trunk’ event already detectable in the premalignant lesion, serous tubal intraepithelial carcinoma (STIC) [9, 10]. *CCNE1* amplification is mutually exclusive with *BRCA* mutations [8, 11] and correlates with cyclin E1 protein overexpression [5, 12]. It is associated with platinum resistance, reduced disease-free survival and poor prognosis [4, 8, 12]. *CCNE1*-amplified tumors often exhibit higher CDK2 expression [13]. Although pharmacological CCNE1 inhibitors are not yet available, CDK2 may be an attractive target for intervention, given the availability of small molecule CDK2 inhibitors. The non-selective CDK inhibitor dinaciclib (SCH-727965) inhibits CDK2 and is currently on trial for hematological malignancies and solid tumors (NCT00798213 and NCT00937937 and [14, 15]). It has been shown to be efficacious in preclinical models of ovarian cancer [16, 17].

As the molecular basis of HGSOC remains poorly understood, little progress has been achieved in the management of the disease during the last 3 decades and mortality rate remains high. New therapeutic possibilities such as anti-angiogenic drugs, PARP inhibitors and immune checkpoint inhibitors are under intense interest [18, 19]. However, predictive biomarkers for these treatments, as well as for cytotoxic drugs, are still missing. Recently, *CCNE1* amplification has been proposed for patient selection in clinical trials of CDK2 inhibitors in HGSOC [5, 17].

HGSOC is commonly being diagnosed at advance stage, as a metastatic disease. To increase the likelihood of optimal surgical debulking, 3 cycles of neoadjuvant chemotherapy (NACT, taxane and platinum doublet) are often administered before surgery, complemented by 3 additional cycles of the same chemotherapy following surgery (adjuvant therapy) [20, 21]. Ideally we would like to personalize the protocol for the adjuvant phase of the treatment, based on the pathological response to NACT, but this trivial clinical need has not yet been incorporated into daily practice.

Böhm *et al.* has recently presented a prognostic significant and reproducible system for grading response of HGSOC to NACT [22]. The system is based on examination of post-surgical derived formalin fixed paraffin embedded (FFPE) samples, and is defined by a three-tier chemotherapy response score (CRS), where CRS1 represents no or minimal tumor response, CRS2 represents appreciable tumor response amid viable tumor that is readily identifiable, and CRS3 represents complete or near-complete response.

Given the reported relative chemoresistance of *CCNE1*-amplified HGSOC tumors, we hypothesized that post-NACT residual tumors may be enriched for CCNE1-overexpressing cells. Furthermore, pre-and post-NACT CCNE1-positivity may serve as a predictive biomarker for lack of benefit from first-line taxane-platinum chemotherapy, suggesting a possible indication for CDK2 inhibition in the adjuvant setting. To test these hypotheses, we assembled a unique collection of well-annotated pre- and post-NACT HGSOC specimens. We compared CCNE1 immunohistochemical staining in matched pre- vs. post-NACT HGSOC tumors, and examined the degree of enrichment of CCNE1-positive cells following NACT. We also correlated CCNE1 intensity in pre-treatment tumors to their CRS scores, to evaluate the utility of CCNE1 as a predictive biomarker for first-line chemosensitivity.

## RESULTS

### CCNE1-overexpressing cell population is not enriched following NACT

**Figure 1:**
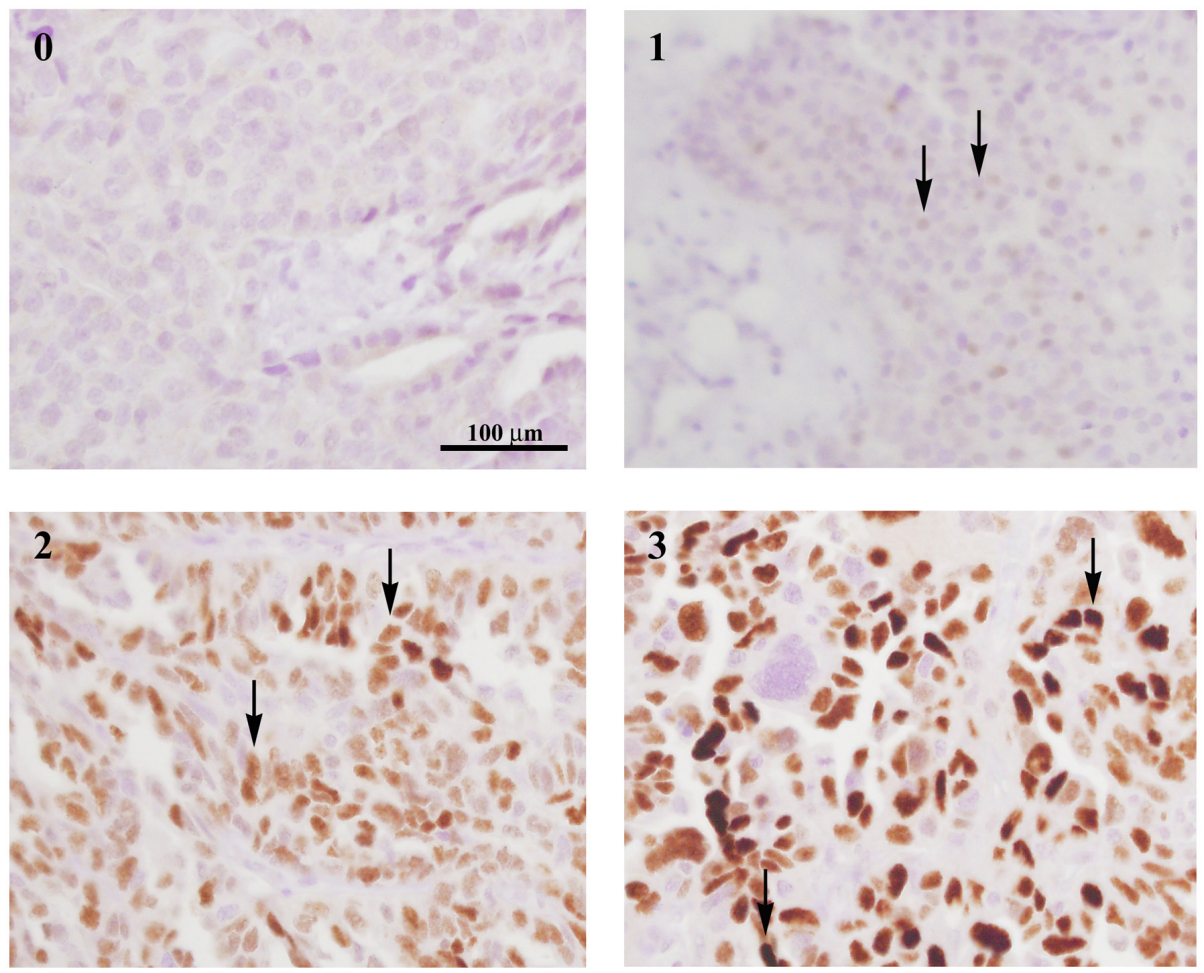
CCNE1 staining intensity spectrum Histological sections of pre-treatment omental core needle biopsies were immuno-stained with CCNE1 antibody. Staining intensity (ranging from 0 to 3) of immuno-positive tumor cells is exemplified as indicated by arrows. All histology images are in X400 magnification.

**Figure 2:**
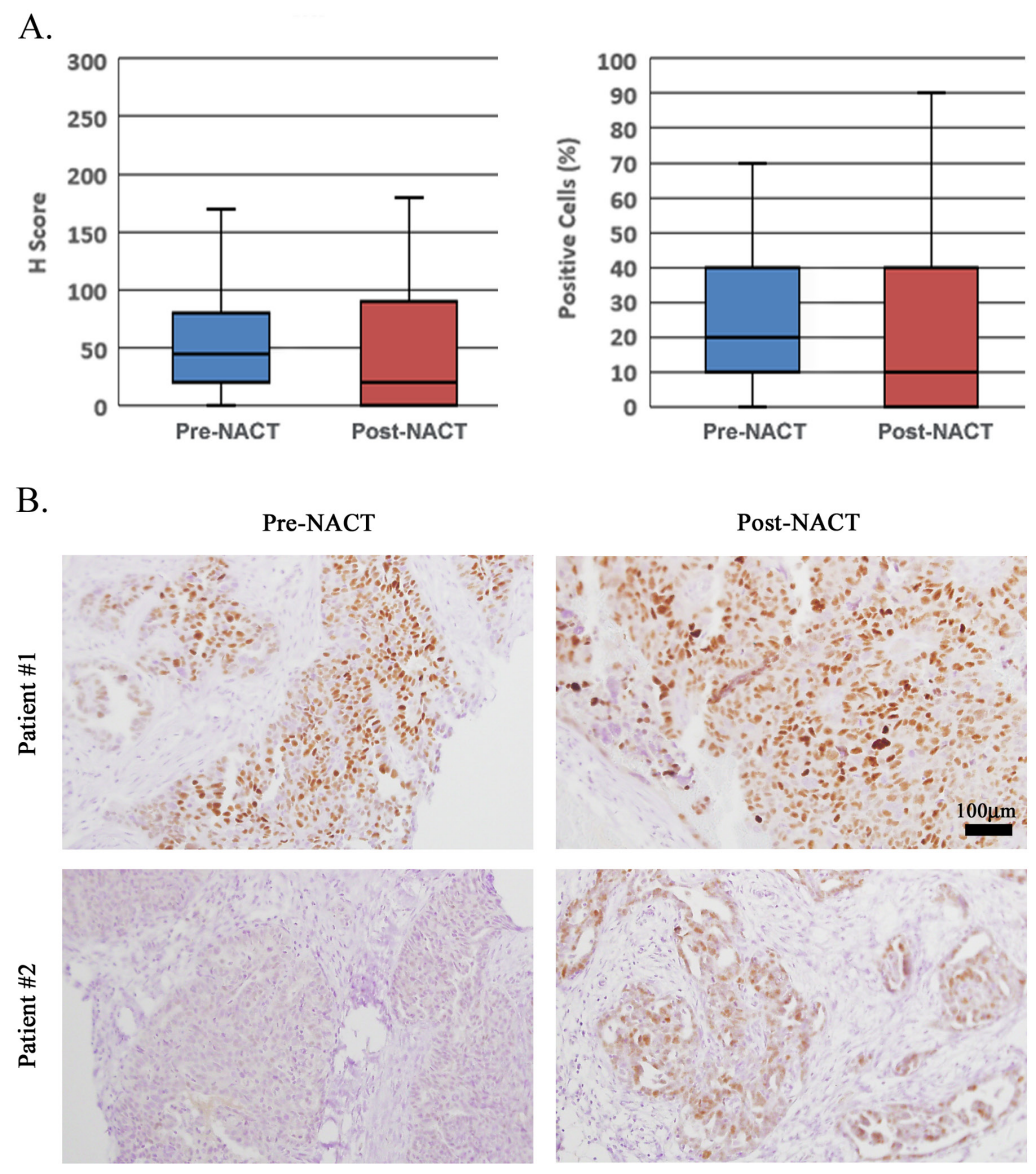
CCNE1-overexpressing cell population is not enriched following NACT **(A)** Matched pre-treatment omental core needle biopsies and post-NACT HGSOC tumors were evaluated for CCNE1 positivity. H-score as well as PC values are presented. **(B)** IHC staining for CCNE1 of matched pre- vs. post-NACT specimens from two different patients showing no CCNE1 enrichment in patient #1 vs. significant enrichment in patient #2. All histology images are in X200 magnification.

Previous clinical data shows that *CCNE1* amplification is associated with platinum resistance and poor prognosis. We therefore aimed to test the hypothesis that platinum-based chemotherapy has greater cytotoxic effect on non *CCNE1*-amplified tumor cells, thus resulting in enrichment for *CCNE1*-amplified cell population, which might underlie and boost disease recurrence. *CCNE1* amplification was previously shown to correlate with CCNE1 overexpression [9]. We therefore examined 19 matched pre- vs. post-NACT HGSOC tumors (for detailed patients’ characteristics see Supplementary Table 1) by immunohistochemical staining for CCNE1. For each sample we calculated the percentage of positive cells (PC) out of the total tumor cells, as well as the H-score. As detailed in the methods section, H-score represents weighted average of staining intensities within the examined field (range 0-300, exemplified in Figure 1). NACT did not result in enrichment for CCNE1-positive cells. Whereas the median H-score and PC values for the pre-NACT biopsies were 45 and 20%, respectively, post-treatment values decreased to 20 and 10%, respectively, however not significantly different (Figure 2A, 2 tails paired *t test*; n=19, p=0.08, p=0.17, respectively). Though in individual cases enrichment for CCNE1-positivity could be observed following NACT (Figure 2B lower panel), the average fold-change for both H-score and PC values was 0.7, indicating a net decrease in CCNE1 staining. We also performed subgroup analysis of the cases which displayed partial or complete pathological response to NACT vs. poor response. Nevertheless, there was no enrichment for CCNE1 in post-NACT tumors, in either subgroup.

To address the concern of different biological behavior of tumors assigned to upfront NACT, we evaluated the CCNE1 H-score and PC in 11 primary debulked HGSOC tumors (Supplementary Table 1). A Kaplan-Meier survival curve for the primary debulking cohort overlaps with that of the NACT cohort (Supplementary Figure 1, Logrank test, p=0.79). No significant difference was observed in terms of CCNE1 positivity between patients who were assigned to primary debulking surgery, compared to pre- or post-NACT specimens (data not shown, p=0.19, p=0.35 respectively).

### Pre-NACT CCNE1 expression does not correlate with pathological response to chemotherapy

**Figure 3:**
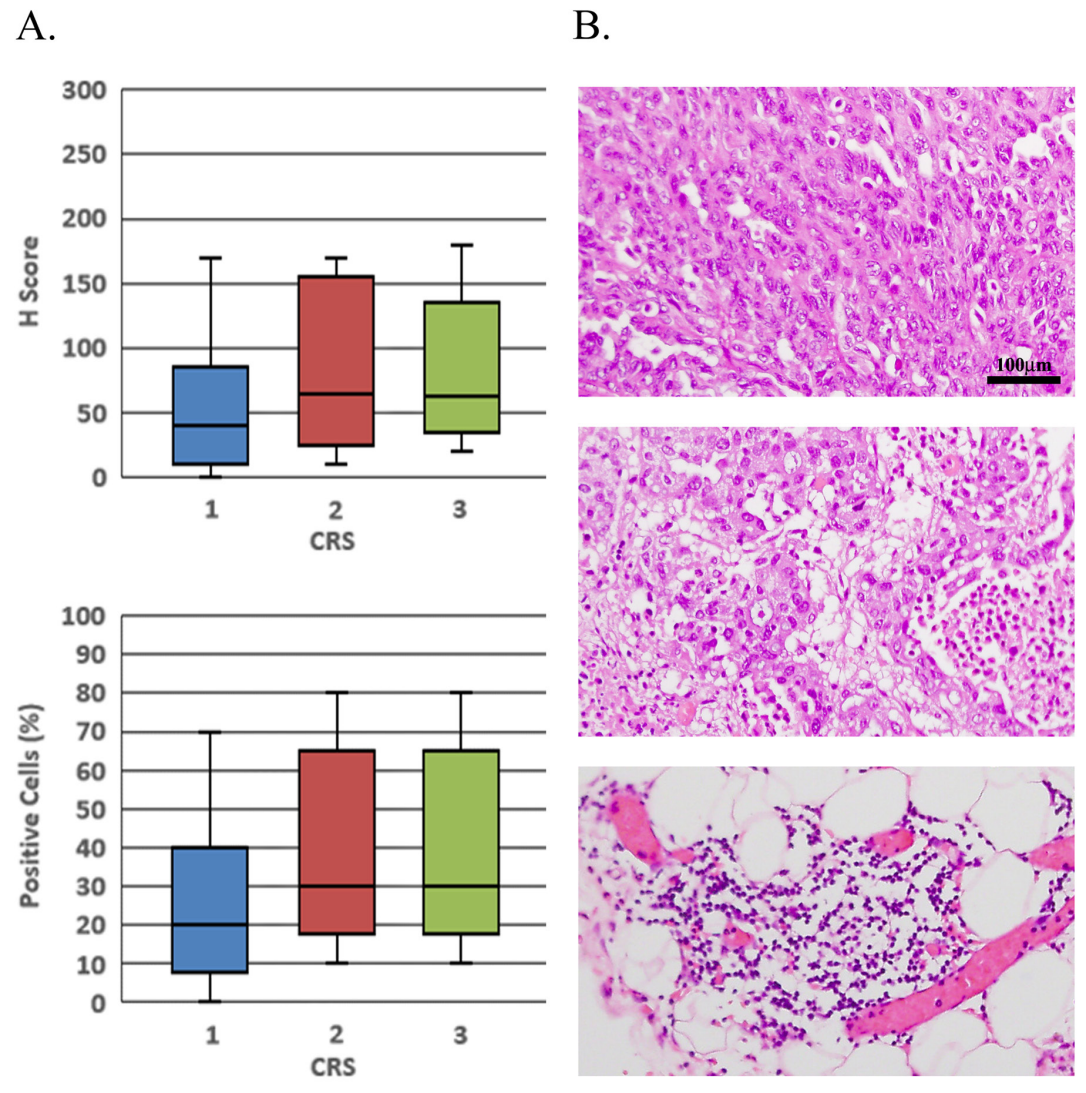
Pre-NACT CCNE1 expression does not correlate with pathological response to chemotherapy **(A)** Pre-treatment omental core needle biopsies were immuno-stained with CCNE1 antibody and CRS was assessed for matched post-NACT HGSOC tumors. H-score as well as PC values are presented as a function of CRS. **(B)** H & E staining of post-NACT HGSOC tumors representing CRS1, 2 and 3 (upper, middle and lower panels respectively). All histology images are in X200 magnification.

Next, we examined the utility of CCNE1 as a predictive biomarker for first-line chemosensitivity. CRS has been recently adopted into the guidelines of pathological evaluation of HGSOC interval debulking specimens, due to its significant correlation with patients’ prognosis [22]. We examined 21 post-NACT omental specimens, searched for residual tumors and correlated the degree of pathological response to NACT with CCNE1 staining on pre-NACT biopsies. The correlation between H-score of pre-NACT biopsies and CRS of residual tumors was calculated. We hypothesized that H-score will inversely correlate with CRS, indicating poor pathological response to NACT in CCNE1-overexpressing tumors. Non-significant, weak positive correlation was observed, with H-score median values of 40, 65 and 62.5 corresponding to CRS1, CRS2 and CRS3, respectively, (Figure 3A top, Pearson correlation, r(20) = 0.22, p=0.17). Similar results were found when pre-NACT PC values were correlated with CRS: CRS1 corresponded to a median 20% CCNE1 positivity, while both CRS2 and CRS3 corresponded to a median 30% CCNE1 positivity (Figure 3A bottom, Pearson correlation, r(20)=0.24, p=0.15). Furthermore, since we concluded that there was no significant difference between the CCNE1 staining intensity in matched pre- and post-NACT specimens, we included in the analysis 8 additional post-NACT HGSOC specimens (Supplementary Table 1) for which we did not have a matched pre-NACT biopsy. The conclusion remains unchanged: the data does not indicate correlation between CCNE1 staining and CRS (r(28)=0.11, p=0.29 for H-score analysis, r(28)=0.08, p=0.33 for PC analysis).

### CCNE1 expression does not correlate with survival

**Figure 4:**
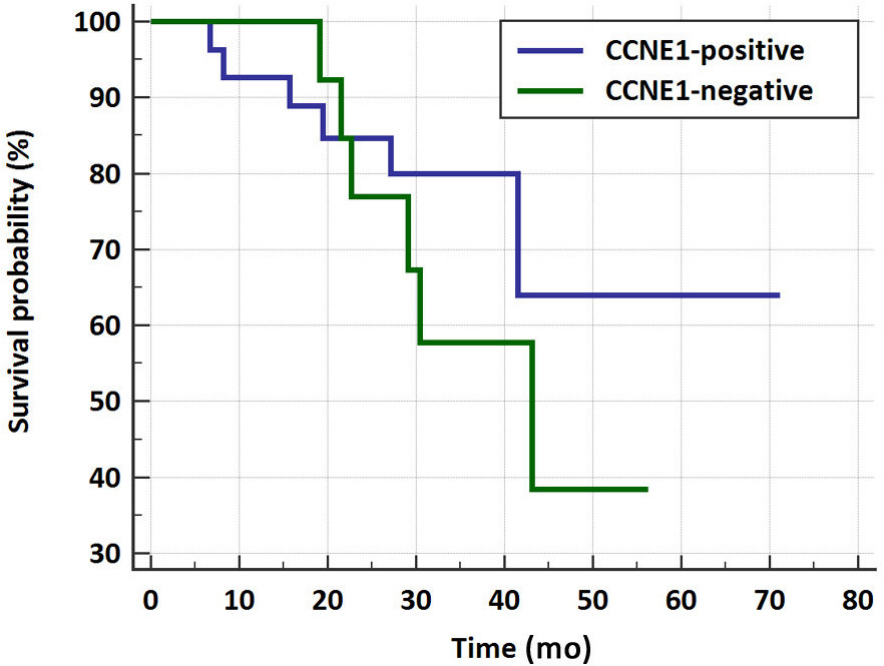
Kaplan-Meier survival curve for CCNE1- positive vs. negative HGSOC patients Overall survival of a total of 40 HGSOC cases was analyzed in view of CCNE1 positivity. Positive cases defined as >10% PC, and negative cases as ≤10% PC (Logrank test, p=0.3). x-axis: time in months.

To directly address the correlation between CCNE1 staining and overall survival, we performed Kaplan-Meier analysis on all 40 cases analyzed for this study (21 cases with matched pre- and post-NACT FFPE specimens, 8 cases with only post-NACT FFPE specimens, and 11 cases with primary debulking specimens). As CCNE1 positivity is a non-dichotomous variable, we considered all cases with >10% PC as CCNE1-positive and ≤10% as CCNE1-negative. Figure 4 indicates that CCNE1 expression does not correspond with overall survival in our cohort (Logrank test, p=0.3).

## DISCUSSION

CCNE1 is a key regulator of cell cycle processes, and genomic amplification of the gene-encoding 19q12-q13 locus occurs in ∼20% of HGSOC as a ‘trunk’ genomic event. It was previously reported that *CCNE1* amplification is associated with platinum resistance, reduced disease-free survival and poor prognosis. The protein was therefore suggested as a target for intervention, and its amplification as a predictive biomarker.

The current study aimed to directly examine the role of CCNE1 positive-immunostain as a predictor of first-line taxane-platinum chemoresistance. The accepted therapeutic protocol with neoadjuvant therapy followed by debulking surgery sequence provides an opportunity to assess the patient’s clinical and pathological response and potentially improve the efficacy of the subsequent adjuvant therapy cycles. However, to date, no guidelines or clinical trials address this challenge. Given the reported relative chemoresistance of *CCNE1*-amplified HGSOC tumors, we hypothesized that post-NACT residual tumors may be enriched for CCNE1-overexpressing cells. CCNE1 enriched tumors may be thereafter targeted by small molecule CDK2 inhibitors, as CCNE1 function depends on CDK2. We therefore evaluated matched pre- vs. post-NACT FFPE samples of HGSOC tumors and correlated the degree of pathological response to CCNE1 expression levels. Our study did not detect CCNE1 enrichment following NACT nor inverse correlation between pathological response (as represented by CRS values) and CCNE1 H-score, thus could not support our hypothesis.

Few possible explanations may clarify our observations. First, our cohort may be too small to express the differences between responders and non-responders. Larger sets of matched pre- and post-NACT specimens are required but not easily attainable. Secondly, CCNE1 amplification may not have a significant influence on the cellular sensitivity to first-line taxane-platinum chemotherapy, but rather correlate with acquired chemoresistance and disease progression at later evolutionary steps only. Thirdly, the amplified genomic locus 19q12-q13, which is correlated with poor prognosis, may exert its effect on the carcinogenic cellular behavior by different mechanism than CCNE1 overexpression. It was previously reported that *URI* [23], which is adjacent to CCNE1 at the 19q12 amplicon, may also contribute to the oncogenic effect. Additionally, the locus may contain yet unrecognized regulatory sequences.

Interestingly, The Cancer Genome Atlas (TCGA) Research Network reported that CCNE1 amplification does not confer survival disadvantage, compared to a purely *BRCA* wild-type cohort. As CCNE1 amplification is mutually exclusive with *BRCA* mutations, it was suggested that the previously reported *CCNE1-*amplification survival disadvantage actually reflects better survival of *BRCA*-mutated cases [8]. Our overall survival results are in line with the TCGA analysis.

In view of the intense effort to discover clinically useful biomarkers, we perceive these essentially negative results as valuable and meaningful. Our results indicate that CCNE1 expression cannot serve as a predictive biomarker for taxane-platinum chemoresistance in the neoadjuvant or adjuvant setting for HGSOC patients. Further research is required in order to enable personalized adjuvant treatment, in cases where poor pathological response is achieved after the neoadjuvant phase.

## MATERIALS AND METHODS

### Case selection

We assembled a set of 21 archival FFPE specimens of matched pre-treatment omental core needle biopsies and post-NACT HGSOC tumors (specimens from debulking surgery, performed after 3 cycles of neoadjuvant paclitaxel-carboplatin). In addition, FFPE specimens of 8 post-NACT HGSOC tumors as well as 11 cases of primary debulked HGSOC were collected. These primary debulked samples were used as a control group. All samples were retrieved from the 2010-2016 archives of the Department of Pathology at Chaim Sheba Medical Center, with appropriate ethical committee approvals. Of note: in the clinical setting, most diagnostic biopsies are cytology specimens. However, we could not use these biopsies for evaluation of CCNE1 expression by immunohistochemistry (IHC). For many samples, there were no available cell-blocks. Furthermore, preliminary survey we performed showed that cytological specimens were negative for CCNE1 IHC staining even when pathological specimens of these tumors stained positively.

### Immunohistochemistry (IHC)

Sections from all FFPE blocks were simultaneously stained with anti-Cyclin E1 mouse monoclonal antibody (ab9517, Abcam, Burlingame, CA, USA) at 1:10 dilution for 1h at 37°C on a Ventana platform (Roche, Tucson, AZ, USA). Matching hematoxylin eosin (H & E)-stained sections were available for all CCNE1-stained sections, to confirm the correct identification of viable tumor cells. Staining intensity (ranging from 0 to 3) of immuno-positive tumor cells was evaluated by a certified pathologist applying routine rules of clinical histopathological examination. For each section the entire tissue area was examined and the percentage of cells at each category was determined. The total percentage of positive cells (PC) was calculated. In addition, an H-score, representing a weighted average of staining intensities (ranging from 0 to 300) was defined for each sample.

### Chemotherapy response score

Matched post-NACT omental specimens stained by hematoxylin-eosin were scored by a certified pathologist according to the 3-tier CRS system for HGSOC [22]. Briefly, CRS1 represents no or minimal tumor response, CRS2 represents appreciable tumor response amid viable tumor that is readily identifiable, and CRS3 represents complete or near complete response.

### Statistical analysis

Statistical significance (p < 0.05) was assessed by t-test, by Pearson correlation or by Logrank test as indicated.

## SUPPLEMENTARY MATERIALS FIGURE AND TABLE





## Abbreviations

CCNE1: Cyclin E1
HGSOC: high grade serous ovarian cancer
STIC: serous tubal intraepithelial carcinoma
NACT: neoadjuvant chemotherapy
CRS: chemotherapy response score
FFPE: formalin fixed paraffin embedded
IHC: immunohistochemistry
PC: positive cells
TCGA: The Cancer Genome Atlas
