# Supplementary material for: CCNE1 expression in high grade serous carcinoma does not correlate with chemoresistance
Source: Oncotarget. 2017 Jul 15;8(37):62240–7. doi: 10.18632/oncotarget.19272 (PMC5617501; doi:10.18632/oncotarget.19272)
Supplement: Supplementary file 1 [file oncotarget-08-62240-s001.pdf]

CCNE1 expression in high grade serous carcinoma does not correlate with chemoresistance

SUPPLEMENTARY MATERIALS

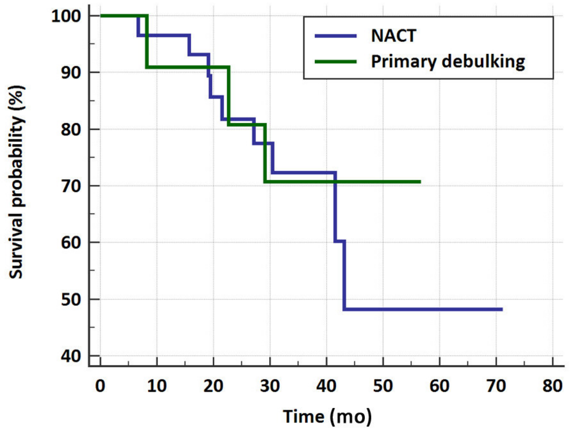

Supplementary Figure 1: Kaplan-Meier survival curve of HGSOC patients undergoing primary debulking vs. NACT followed by interval debulking. Survival of 29 cases of NACT and 11 cases of primary debulked patients whose tumor specimens were analyzed for CCNE1 is plotted (Logrank test, p=0.79). x-axis: time in months.

Supplementary Table 1: Patients' characteristics.

See Supplementary File 1
